# Candidiasis and Other Bacterial Infections among Patients Diagnosed with Burning Mouth Syndrome

**DOI:** 10.3390/medicina58081029

**Published:** 2022-08-01

**Authors:** Viktors Jankovskis, Guntars Selga

**Affiliations:** Department of Oral Medicine, RSU Institute of Stomatology, Rīga Stradiņš University, LV-1007 Riga, Latvia; selga@sveiks.lv

**Keywords:** glossodynia, burning mouth syndrome, candidiasis, bacterial infection

## Abstract

*Background and Objectives:* Burning mouth syndrome (BMS) is a state in which a patient experiences intraoral burning or a dysesthetic sensation without clinically evident causative lesions in the oropharyngeal area. The disorder is linked to a variety of conditions, including dry mouth, *Candida*, and bacterial infections. The aim of this study was to determine the incidence of oral *Candida* and/or bacterial infections among patients with BMS and whether they have an effect on pain/burning and salivary flow levels. Objectives: (1) Gather patient data regarding the presence of oral infections, dry mouth, and pain levels in the morning, afternoon, and evening periods; (2) data analysis and assessment to determine medians, means, frequencies, correlations, and statistically significant differences between patient groups. *Materials and Methods*: Overall, 173 patients (23 males and 150 females) with BMS and 13 controls (five males and eight females) took part in the study. We measured pain/burning levels, unstimulated and stimulated salivary flow, the percentage of patients infected with *Candida* species and/or bacterial species, and the said species growth in Petri dishes. *Results*: *Candida albicans* was the most commonly found infection among patients with BMS (*n* = 28, 16.2%). Overall, 21.4% patients with BMS were diagnosed with either *C. albicans* or another *Candida* species. *Enterobacter* had the richest growth among patients with BMS (7.5% out of the infected 10.4% BMS patients). No statistical significance could be noted between the existence of either *Candida* species or bacterial species infections and changes in pain/burning and salivary flow levels. Negative correlations were noted between age and unstimulated and stimulated salivary flow, and positive correlations were noted between age and *Candida* andspecific bacteria species’ growth levels. *Conclusions*: Although patients with present bacterial or *Candida* infections showed a marginal increase in pain/burning levels, no direct statistically significant associations could be made between the presence of *Candida* species or other bacteria and the symptoms among patients with BMS.

## 1. Introduction

Burning mouth syndrome (BMS) is a state in which a patient experiences an intraoral burning or dysesthetic sensation without clinically evident causative lesions in the oropharyngeal area. An additional and helpful, but not mandatory, diagnostic criteria can be the chronicity, in which the state of being symptomatic is persistent (typically >3 months) [1,2,3]. A patient may experience a triad of symptoms—a burning sensation, dysgeusia, and xerostomia—but for a patient to experience all three of the symptoms at the same time is a rarity [4]. The pain condition is likely neuropathic, mediated by varied levels of dysfunction in the peripheral and central nervous system. It largely affects perimenopausal and postmenopausal women. It is diagnosed after a thorough history and clinical examination and indicated laboratory studies to rule out a local or systemic cause, which is treated accordingly after being identified [5]. BMS affects 15% of adults overall [3]. The disorder has long been linked to a variety of conditions: menopause, diabetes, nutritional deficiencies, disorders of the mouth, dry mouth, acid reflux, and cancer therapy, with candidiasis and psychological disorders having a significant association with the occurrence of BMS [6,7,8].

In one study, the oral carriage of *Candida* was found in more than 50% of patients with oral dysesthesia [9]. Patients with BMS may also have the presence of non-oral bacteria in the oral cavity, which can have an effect on the host. Oral bacterial, viral, and/or fungal infections have been considered as local factors involved in causing BMS [10,11].

Older female patients with BMS exhibit a significant reduction in unstimulated whole saliva, as well as a higher prevalence of xerostomia and gastrointestinal diseases. Hyposalivation is a risk factor for poorer oral health, especially in tongue coating and gingival inflammation and oral *Candida* colonization among patients. Salivary flow rates can be further decreased by medication usage. Candidiasis in conjunction with hyposalivation may induce pain in the tongue without the manifestation of objective abnormalities [3,12,13,14].

The complete remission of BMS occurs only in a small percentage of patients. Up to 30% will note moderate improvement with or without treatment [3].

The aim of this study was to determine the incidence of oral *Candida* or/and bacterial infections among patients with BMS and whether they have an effect on pain/burning and salivary flow levels.

The objectives were as follows:

(1) To gather patient data regarding the presence of oral infections, dry mouth, and pain levels in the morning, afternoon, and evening periods;
(2) To conduct data analysis and assessments to determine the medians, means, frequencies, correlations, and statistically significant differences between patient groups.

## 2. Materials and Methods

The research data were acquired between 31 October 2018 and 6 April 2022. The clinical trial was conducted at the Institute of Stomatology (Stomatoloģijas Institūts), Riga, Latvia. This controlled clinical trial was part of a doctoral thesis, which was approved by the Research Ethics Committee of Rīga Stradiņš University (RSU). The research was conducted in accordance with the Helsinki Declaration. All subjects gave their informed consent for inclusion in the study. Overall, 173 patients (23 males and 150 females) and 13 controls (5 males and 8 females) took part in the study. The control group of patients was provided by the Prosthodontics Department. The patient and control groups were relatively small due to the COVID-19 restrictions imposed throughout 2020–2022 and a general lack of willingness among patients to take part in such studies during the pandemic.

### 2.1. Inclusion Criteria for BMS Patients

- Any type of oropharyngeal symptom (specifically, burning or pain sensations) that can be persistent or intermittent with possible phases of remission/exacerbation during the day;
- The absence of any clinically and instrumentally detectable oropharyngeal lesion;
- The absence of any type of local and/or systemic factor, such as oral diseases, drugs (for instance, antibiotics), trauma, hypersensitivity reactions, or physical/chemical agents;
- In addition (but not mandatory): symptoms are persistent (typically 3 months) [2].

### 2.2. Inclusion Criteria for Control Group

- The absence of any oral pathology that is associated with oral lesions;
- The absence of any clinically and instrumentally detectable oropharyngeal lesion;
- The absence of any type of local and/or systemic factor, such as oral diseases, drugs (for instance, antibiotics), trauma, hypersensitivity reactions, or physical/chemical agents.

### 2.3. Assessment Method

To determine the median pain/burning sensation’s intensity, a visual analogue scale (VAS) was used [15]. Patients received a scale from 0–10, and were required to tick their pain/burning sensation intensity levels for the morning, afternoon, and evening periods. Pain/burning levels were determined and compared in the BMS patient group between the infected and non-infected overall and where infection rates in the BMS group exceeded more than 5% for specific pathogens. Pain/burning levels in the BMS group were compared between genders and age groups (ages below 50 years, and ages of 50 years and above).

During the study, patients’ median salivary flow levels were determined. Patients were required to collect saliva into 2 tubes (one for unstimulated saliva, and one for stimulated saliva) for 5 min each. The flow of the unstimulated saliva was observed when the patient was at rest. The stimulated saliva flow was collected after the patient chewed a piece of paraffin to stimulate salivation. The norm was set at 0.2 mL/min for unstimulated saliva flow, and 0.5–2 mL/min for stimulated saliva flow. If patients had an unstimulated salivary flow of <0.2 mL/min and a stimulated flow <0.5 mL/min, it was confirmed that the patient had hyposalivation. The salivary flow levels were determined during patient visits from 11:00 until 15:30. Salivary flow levels were determined and compared in the BMS patient group and control patient group between the infected and not infected patients overall and where infection rates in the BMS group exceeded more than 5% for specific pathogens, and in the control group, where the infection rates affected more than one patient. Additionally, the salivary flow levels in the BMS group and the control group were compared between genders and age groups (ages below 50 years, and ages of 50 years and above) [16].

To determine the presence of *Candida* and bacterial species, a cotton swab test was performed on the dorsal surface of the tongue’s body. The cotton swab was then placed into a sealed tube to be delivered to a certified clinical laboratory. The type of pathogen was determined, as well as its growth levels (poor, medium, rich). In our study, we expected that the most often found pathogen would be *Candida*, as well as *Klebsiella species, S. aureus,* and *Enterobacter species*, since those have been found in considerable amounts in patients with BMS in previous research [6,7,8,10,11]. The test was performed for BMS group patients and control group patients to determine the infection rates and growth levels.

At the laboratory, the growth amount was determined through the colony count that had grown in a Petri dish:

- Poor—less than 10 colonies per Petri dish;
- Medium—10 to 100 colonies per Petri dish; and
- Rich—more than 100 colonies per Petri dish.

Poor and medium growth indicated that the patient was a carrier of the pathogen, but rich growth presented an infection.

The duration of disease was determined and compared in the BMS patient group between the infected and non-infected overall and where infection rates in the BMS group exceeded more than 5% for specific pathogens. Pain/burning levels in the BMS group were compared between genders and age groups (ages below 50 years, and ages of 50 years and above).

Additionally, other parameters were also determined—the median and mean age and sex of the patients, as well as the most affected area.

### 2.4. Statistical Analysis

Data were analyzed using the Statistical Package for Social Sciences (SPSS for Windows, version 23.0, SPSS Inc., Chicago, IL, USA). Since practically none of the data had a normal distribution, the Mann–Whitney U test (for nonparametric data) was used for variable comparisons between groups (age groups, genders, and BMS and control groups). The adopted significance level was 5% (*p* < 0.05). Overall patient data and data frequencies were also checked.

To check correlations, the Spearman correlation test was used between patient age, gender, pain levels in the morning, pain levels in the afternoon, pain levels in the evening, unstimulated salivary flow levels, stimulated salivary flow levels, *Candida albicans* growth levels, *Candida species* growth levels, *Staphylococcus aureus* growth levels, *Serratia marcescens* growth levels, *Aeromonas* species growth levels, *Acinetobacter lwoffi* growth levels, non-fermenting Gram-negative rod growth levels, *Klebsiella pneumoniae* growth levels, *Citrobacter* species growth levels, *Pseudomonas* species growth levels, *Klebsiella oxytoca* growth levels, *Escherichia coli* growth levels, *Acinetobacter ursungii* growth levels, *Serratia* species growth levels, *Enterobacter* species growth levels, *Acinetobacter junii* growth levels, *Serratia ficaria* growth levels, *Citrobacter freundii* growth levels, *Raoutella ornithinolytica* growth levels, *Acinetobacter baumanii* growth levels, *Klebsiella* species growth levels, *Pseudomonas putida* growth levels, *Comamonas testosteroni* growth levels, *Raoutella planticola* growth levels, *Burkholderia gladioli* growth levels, *Pantoea* species growth levels, *Pseudomonas fluorescens* growth levels, *Sphingomonas paucimobillis* growth levels, *Rahnella aqualitis* growth levels, *Moraxella* species growth levels, *Morganella morganii* growth levels, *Pseudomonas aeruginosa* growth levels, *Enterobacter asburiae* growth levels, *Stenotrophomonas maltophilia* growth levels, *Proteus vulgaris* growth levels, and duration of disease.

## 3. Results

### 3.1. Overall BMS Patient Group and Control Patient Group Data

#### 3.1.1. Age

The median age for 173 of the BMS patients in the study was 57 (interquartile range (IQR 19)) years, and the mean age was 56.8 ± 14.03 years. A total of 51 patients (29.5%) were below the age of 50, and 122 patients (70.5%) were in the age group of 50 and above.

The median age for 13 of the control group patients in the study was 63 (IQR 17) years. The mean age was 59.77 ± 10.96 years.

#### 3.1.2. Affected Areas

The most affected area by the burning sensation was the tongue, affecting 170 patients. Three patients had no burning of the tongue itself.

Seventy-nine patients had burning in other parts of the oral mucosa (palate, cheeks, floor of the mouth, lips, gingiva), and 94 patients had no burning symptoms in other parts of the oral mucosa.

#### 3.1.3. Duration of Disease

The median duration of disease was 5 (IQR 9) months. The mean duration was 11.87 ± 21.30 months.

Females had the same duration as mentioned previously, but males had a median duration of 6 (IQR 9) months. Overall, no statistical difference was noted (z = −0.21, *p* = 0.83).

Age groups below 50 years had a median duration of 5 (IQR 9) months. The same median duration was observed for the BMS patients above the age of 50 years. Overall, no statistical difference was noted (z = −0.69, *p* = 0.49).

### 3.2. Pain/Burning Levels for BMS Patients

#### 3.2.1. Morning

The median pain/burning sensation levels in the morning had a score of 2 (IQR 2). Female patients had a pain/burning median score of 2 (IQR 2), and the male median score was 2 (IQR 3). The data difference between male and female BMS patients was not statistically significant (z = −0.14, *p* = 0.89).

The group aged below 50 years had a median score of 2 (IQR 2). Patients aged 50 years and above had a median score of 2 (IQR 2). The data difference between the age groups was not statistically significant (z = −0.79, *p* = 0.43).

#### 3.2.2. Afternoon

Median pain/burning sensation levels in the afternoon had a score of 4 (IQR 2). The female patients had a pain/burning median score of 4 (IQR 2), and the male median score was also 4 (IQR 3). The data difference between male and female BMS patients was not statistically significant (z = −1.41, *p* = 0.16).

Age groups below 50 years had a median score of 4 (IQR 2). Patients aged 50 years and above had a median score of 4 (IQR 1.6). The data difference between the age groups was statistically significant (z = −1.89, *p* = 0.06).

#### 3.2.3. Evening

The median pain/burning sensation levels in the evening had a score of 5.5 (IQR 3). Female patients had a pain/burning median score of 5.75 (IQR 3), and the male median score was 4 (IQR 2). The data difference between male and female BMS patients was not statistically significant (z = −0.83, *p* = 0.41).

Age groups below 50 years had a median score of 6 (IQR 3). Patients aged 50 years and above had a median score of 5 (IQR 3). The data difference between the age groups was not statistically significant (z = −0.93, *p* = 0.35).

#### 3.2.4. Overall Pain Data

The data showed an increase in the pain/burning sensation throughout the day from morning until evening, with scores rising from 2 to 5.5. None of the pain data were statistically different either between the age groups or the genders of the patients.

### 3.3. Pathogen Counts among BMS Group Patients and Control Group Patients

#### 3.3.1. Percentage (%) of BMS Patients Affected by a Pathogen and Pathogen Growth Rate Percentages That Were in the BMS Group

Infection affected 63 (36.4%) of the 173 BMS group patients. Table 1 shows the number of patients affected by each pathogen.

The most frequent infectious diseases were *Candida albicans*, *Staphylococcus aureus*, *Enterobacter*, *Klebsiella pneumoniae*, non-fermenting Gram-negative rods, *Klebsiella oxytoca*, *Candida* species, and *Escherichia coli*, constituting more than 5% of infections, as shown in Table 1.

Patients with *Enterobacter, Klebsiella pneumoniae*, and non-fermenting Gram-negative rods had the highest number of cases with rich pathogen growth levels, as shown in Table 2.

#### 3.3.2. Percentage (%) of Control Group Patients Affected by a Pathogen and Pathogen Growth Rate Percentages That Were in the Control Group

Infection affected 8 (61.5%) of the 13 control group patients. Table 1 shows the number of patients affected by each pathogen. Some patients had more than one pathogen.

The most frequent infectious diseases *were S.aureus, Klebsiella species,* and *Candida albicans,* exceeding 7.7%, as shown in in Table 1.

Only one patient with *A.baumanii* presented a rich growth rate, as shown in Table 2.

#### 3.3.3. Candida Species Infections

*C. albicans* was the most common cause of infection among patients initially diagnosed with BMS, affecting 16.2% of the patients. Additionally, 5.2% of patients were diagnosed with a variety of *Candida* genus species. Overall, 21.4% were diagnosed with either *C. albicans* or another *Candida* species. Out of the 16.2% of patients, only 1.7% had rich growth levels of *C. albicans*; the rest had medium and poor growth levels. Similar results were obtained for *Candida*-species-affected patients, where 1.2% of the affected 5.2% had rich growth levels.

In the control group, 23.1% of patients were affected by *C. albicans* infection. Out of those, two had poor growth levels and one had medium growth levels.

#### 3.3.4. Staphylococcus Aureus Infections

*S. aureus* was the second most common cause of infection among patients initially diagnosed with BMS, affecting 13.9% of the patients. Out of this 13.9% of patients, only 1.2% had rich growth levels of *S. aureus*; the rest had medium and poor growth levels. A statistically significant low negative correlation was noted between the *S. aureus* growth rate and the age of BMS patients.

In the control group, 30.77% (*n* = 4) of patients were affected by *S. aureus*. All had poor growth levels.

#### 3.3.5. Klebsiella Species Infections

*K. pneumoniae* was the most common cause for *Klebsiella* infection among patients with BMS, affecting 8.7% of the patients. The overall percentage of patients that had *Klebsiella* species infections would be 18.5%, since patients did not overlap. Out of the 8.7% of patients, only 4.6% had a rich growth of *K. pneumoniae;* the rest had medium and poor growth. Furthermore, 1.7% of *Klebsiella*-species-affected patients had rich growth levels, and 2.3% of *Klebsiella oxytoca*-affected patients had rich growth.

Among the control group, 15.1% (*n* = 2) had *Klebsiella* species infections. Out of those, one had poor growth and one had medium growth.

#### 3.3.6. Enterobacter Species Infections

Among patients diagnosed initially with BMS, 10.4% were affected by *Enterobacter*. *Enterobacter* had the highest rich growth levels, at 7.5%, compared to all other pathogens. We found that 0.6% (one patient) was infected by *Enterobacter asburiae*, showing rich growth levels.

None of the control patients were affected by *Enterococcus* infections.

#### 3.3.7. Non-Fermenting Gram-Negative Rods Infections

Among patients diagnosed initially with BMS, 6.9% were affected by non-specific non-fermenting Gram-negative (Gr−) rods, with 4% showing rich growth levels. Combining non-specific findings with specific pathogens, Gram-negative rods (*Acinotbacter lwoffi*, *A. Baumanii*, *A. ursungii*, *P. fluorescenesc*, *Pseudomonas* species, *A. junii*, *P. putida*, *B. gladioli*, *Moraxella* sp., *P. aeroginosa,* and *Stenotrophomonas maltophili*) showed a combined percentage of 15.6% affected, with 10.3% having rich growth levels (taking into account the fact that some patients had multiple Gram-negative pathogens present in the same test).

*Acinetobacter lwoffi* and *Acinetobacter baumanii* affected 1.7% of patients, respectively, but *Acinetobacter ursungi* affected 1.2% and *Acinetobacter junii* affected 0.6% of BMS patients. Altogether, 5.2% of patients with BMS were affected by *Acinetobacter* species.

We observed 1.2% *A. lwoffi*-affected patients, 1.2% *A. baumanii*-affected patients, and 0.6% of patients with *A. ursungii* presenting rich growth levels. The rest of the patients had either poor or medium growth levels.

We found that 1.2% of BMS patients had *P. fluorescens*, 0.6% had *Pseudomonas* species, 0.6% had *Pseudomonas putida*, and 0.6% had *P. aeroginosa* infections. All cases had rich growth.

In the control group, only one patient showed the presence of *A. baumanii*, with this being the only pathogen to have rich growth, and one had *Stenotrophomonas maltophili*, with medium growth.

#### 3.3.8. Escherichia Coli Infections

*E. coli* affected 5.2% of BMS patients. Out of the 5.2% of patients, only 3.5% had a rich growth of *E. coli;* the rest had medium or poor growth.

None of the control group patients had *E. coli* infections.

#### 3.3.9. Other Infections (Pathogens Constituted Less Than 5% of the Affected Patients)

*Citrobacter species infections.*

Three (1.7%) BMS patients were infected with *Citrobacter* species, and two (1.2%) were infected with *Citrobacter freundii*. We found that 1.2% out of 1.7% BMS patients that were infected with *Citrobacter* species had rich growth levels. Both patients infected with *Citrobacter freundii* had rich growth levels. *Citrobacter freundii* and *Citrobacter* species’ growth levels showed a positive correlation with age. No cases were noted in the control group.

*Aeromonas species infections.*

Two (1.2%) BMS patients were infected with *Aeromonas* species. Of those, 0.6% had rich growth levels. No cases were reported in the control group.

*Raoutella species infections.*

One (0.6%) BMS patient was infected with *Raoutella ornithinolytica*, and two (1.2%) BMS patients were infected with *Raoultella planticola*. All patients had rich bacterial growth levels. A positive correlation was noted between age and *Rauotella planticola* growth. No cases were reported in the control group.

*Comamonas species infections.*

One (0.6%) BMS patient was infected with *Comamonas testosterone*, showing rich growth levels. No cases were reported in the control group.

*Pantoea species infections.*

One (0.6%) BMS patient had *Pantoea* species infection. The patient reported rich growth levels of the pathogen.

One (7.7%) patient in the control group also had *Pantoea* infection, showing medium growth levels.

### 3.4. Salivary Flow Levels for BMS Group Patients and Control Group Patients

BMS patients’ median salivary flow rate for unstimulated saliva was 0.2 (IQR 0.25) mL/min, and for stimulated saliva, it was 0.9 (IQR 0.72) mL/min. Both flow rates were within the norm.

The unstimulated salivary flow rate median for female BMS patients was 0.2 (IQR 0.21) mL/min, but for males it was 0.3 (IQR 0.38) mL/min. The stimulated salivary flow rate median for female BMS patients was 0.88 (IQR 0.68) mL/min, but for males, it was 1.2 (IQR 0.96) mL/min. The Mann–Whitney U test showed a statistically significant difference in the unstimulated salivary flow (z = −2.94, *p* = 0.03), but not in the stimulated salivary flow (z = −2.50, *p* = 0.12) between genders.

For BMS patients below 50 years of age, the median unstimulated salivary flow rate was 0.28 (IQR 0.26) mL/min, but the stimulated salivary flow rate was 1.0 (IQR 0.96) mL/min. For patients above 50 years of age, the median unstimulated salivary flow rate was 0.17 (IQR 0.17) mL/min, but the stimulated salivary flow rate was 0.86 (IQR 0.72) mL/min. The Mann–Whitney U test showed a statistically significant difference in the unstimulated salivary flow (z = −3.24, *p* = 0.01) and in the stimulated salivary flow (z = −2.30, *p* = 0.02) between both age groups.

For the control group, the median unstimulated salivary flow rate was 0.32 (IQR 0.18) mL/min, and the stimulated salivary flow rate was 1.2 (IQR 1.19) mL/min. For the female patient group (*n* = 5), the unstimulated salivary flow rate was 0.32 (IQR 0.25) mL/min, but for the male patient group (*n* = 8), the unstimulated salivary flow rate was 0.35 (IQR 0.63) mL/min, showing no statistically significant differences between the groups overall (z = −0.59 *p* = 0.62). For the female group, the stimulated salivary flow rate was 1.1 (IQR 1.36) mL/min, but for the male group, stimulated salivary flow rate was 1.5 (IQR 0.85) mL/min, showing no statistically significant differences between the groups overall (z = −0.88 *p* = 0.44).

For controls patients below 50 years of age (*n* = 3), the median unstimulated salivary flow rate was 0.22 (IQR 0) mL/min, but the stimulated salivary flow rate was 0.52 (IQR 0) mL/min. For patients aged 50 years and above (*n* = 10), the median unstimulated salivary flow rate was 0.34 (IQR 0.16) mL/min, but the stimulated salivary flow rate was 1.2 (IQR 0.90) mL/min. The Mann–Whitney U test showed no statistically significant difference in the unstimulated salivary flow (z = −0.85, *p* = 0.47) and in the stimulated salivary flow (z = −0.85, *p* = 0.47) between both age groups.

### 3.5. Differences of Pain Levels, Salivary Flow Levels, and Duration of Disease between Infected and Non-Infected BMS Groups

Table 3 presents data regarding the differences between infected (by any pathogen) and non-infected BMS groups, and between those that were and were not affected by *Candida albicans*.

None of the factors were statistically different between the infected and non-infected groups. None of the factors were statistically different between the *C. albicans*-infected group and the *Candida albicans*-non-infected group.

The rest of the pathogens that exceeded 5% of infection rates were also compared between the infected and non-infected BMS groups. The tendencies were similar to the ones present in Table 1, but a statistically significant difference was noted between the patients infected with Candida species and non-infected patients regarding pain levels for the afternoon period (z = −2.12, *p* = 0.03), as seen in Table S1.

### 3.6. Correlations between BMS Group Patient Data

Correlations were noted between age and unstimulated salivary flow (r = −0.23, *p* = 0.002), stimulated salivary flow (r = −2.36, *p* = 0.002), *Candida species* growth levels (r = 0.22, *p* = 0.04), *Citrobacter freundii* growth levels (r = 0.17, *p* = 0.049), non-fermenting Gram-negative rods’ growth levels (r = 0.16, *p* = 0.04), *Raoutella planticola* growth levels (r = 0.16, *p* = 0.04), *S. aureus* growth levels (r = −0.18, *p* = 0.18), and *Serratia marcescens* growth levels (r = −0.16, *p* = 0.034). They were also noted for:

Pain levels in the afternoon and *Candida* species growth levels (r = 0.16, *p* = 0.04);

Pain levels in the morning and *Citrobacter* species growth levels (r = 0.16, *p* = 0.04);

Stimulated salivary flow levels and *Raoutella planticola* growth levels (r = −0.17, *p* = 0.03);

Duration of disease and *Citrobacter freundii* growth levels (r = 0.16, *p* = 0.03).

The remaining correlation data for BMS patients are accessible in Table S2.

### 3.7. Differences of Pain Levels, Salivary Flow Levels, and Duration of Disease between Infected and Non-Infected Control Groups, Correlations between Control Group Patient Data

No statistically significant differences were noted in the control groups, as shown partially in Table 4 and fully in Table S4. No correlations within the control group were noted, as shown in Table S3.

### 3.8. Comparison between Overall BMS Patient Group and Control Patient Group

Mann–Whitney U tests were also conducted between the BMS group and the control group regarding unstimulated and stimulated salivary flow levels. Statistical significance was noted regarding unstimulated salivary flow levels (z= −2.88, *p* = 0.04). Statistical significance was noted between the groups regarding *Pantoea* species (z = −2.37, *p* = 0.02) and *Stenotrophomonas maltophilia* (z = −2.37, *p* = 0.02).

## 4. Discussion

### 4.1. Overall

BMS has a negative impact on health-related quality of life and oral-health-related quality of life [17]. Age and sex are the main factors involved in the onset of BMS, but psychological disorders and oral infections, such as candidiasis, are associated with its occurrence [6,7,18]. Patients with BMS may have hyposalivation, which is a risk factor for poorer oral health, especially in tongue coating and gingival inflammation, and oral *Candida* colonization among patients. Salivary flow rates and a decreased concentration of secretory immunoglobulin A (SIgA) can be further decreased by medication usage, lack of exercise, and a reduced serum albumin level [3,9,12,13,19,20]. Salivary immune factors repress opportunistic respiratory pathogens by exhibiting agglutination or direct antimicrobial activity [20]. In our study, in the BMS patient group, we were able to find a negative correlation between age and unstimulated and stimulated salivary flow levels, and a positive correlation between age and specific pathogens growth levels (*Candida species*, *Citrobacter freundii*, *Gr negative rods*, *S.aureus*, *Serratia Marcescens*). Moreover, a negative correlation was noted between stimulated salivary flow levels and *Raoutella planticola* growth levels. Statistically significant differences were noted between the BMS group and control group in regards to the unstimulated salivary flow levels, where BMS patients showed lower results, indicating an association between BMS and unstimulated salivary flow levels.

Changes in the oral environment can disrupt the natural balance of the microflora, giving rise to pathogens. The environment of the host determines the composition and gene expression of the resident microbiota [21]. For instance, opportunistic respiratory pathogens have been found in the oral cavities of bedridden elderly patients, suggesting that oral biofilms are reservoirs for the pathogens. There are three factors that determine this: decreased oral immunity, decreased oral commensal bacteria, and the maturation of oral biofilm formation [20].

Antimicrobial-resistant ESKAPE (*Enterococcus faecium*, *Staphylococcus aureus*, *Klebsiella pneumoniae*, *Acinetobacter baumanii*, *Pseudomonas aeruginosa*, and *Enterobacter* species) have genes that provide resistance to antibiotics, making them a global threat to human health. Due to antibacterial drug resistance, collaborative endeavors will require sustainable management practices to reduce the inappropriate use of antibiotics in both the human health and agricultural sectors [22].

### 4.2. Candida Species Infections

In our study, *C. Albicans* was the most common cause of infection among patients initially diagnosed with BMS, affecting 16.2% of the patients and 23.1% of the control.

No statistically significant differences could be found between the *C. albicans*-infected and non-infected groups, but a statistically significant positive correlation was noted between the *Candida* species patients and the age of patients.

The only statistically significant difference between the *Candida*-species-genus-affected and non-affected BMS pain group was in pain levels during the afternoon, where the median of the unaffected group overall was larger, at 4 (IQR 2), than for the affected group, at 2 (IQR 2.5), with a statistical significance of z = −0.742, *p* = 0.034. Although statistically insignificant, similar results were noted for pain levels during the evening, but for the morning period, both groups had identical results. This could be explained by the fact that the group affected by multiple genera of *Candida* species numbered only nine people.

Overall salivary flow levels were lower for patients with BMS compared to controls who had and did not have *C. albicans* infections.

Usually, *Candida* infection presents itself as a white or red patch with soreness and difficulty swallowing, but it can also show itself without noticeable visual symptoms [23,24].

Some authors have pointed out that oral infections caused by various microorganisms have been associated with BMS, particularly *Candida albicans* [7,18,25]. Other authors, however, do not see an association between the presence or load of *Candida* and the diagnosis of burning mouth syndrome/oral dysesthesia [9,26].

Apart from candida-induced burning, it remains unclear if bacterial or viral infections can induce oral burning [27].

Oral candidiasis is mainly caused by *C. albicans*. Around 30% to 50% of the population carry this organism, with the carriage rate increasing with age [23]. Research has shown that the amount of patients with *Candida* who also have BMS can reach up to 63.3%, and patients with a higher level of xerostomia had a larger carriage of Candida [9]. In our study, *C. albicans* infection rates among patient groups were even less than those mentioned in the literature.

Although *C. albicans* was the most common cause of candidiasis among patients with BMS, other forms of *Candida* were also noted, such as *C. parapsilosis*, *C. tropicalis*, *C. krusei*, and *C. kefyr.* The study itself did not conclude that there was a direct association between BMS and *Candida* [26].

Candidiasis can also be caused by *C. glabarata*, *C. guillermondii*, *C. pseudotropicalis*, and *C. stellatoidea*. The manifestations can vary from mild superficial infections to fatal disseminating diseases [23].

Research has shown that *Candida* itself negatively affects the stimulated whole salivary flow rate, and that reduced unstimulated salivary flow levels are a risk factor for “morphologically normal symptomatic candidiasis” and erythematous candidiasis, meaning that they cause each other [24,28]. Increased *C. albicans*, among other pathogens, is also related to periodontal inflammation and tissue destruction [29].

To differentiate between candidiasis and BMS, the literature mentions that spontaneous pain or pain alleviation by stimulation are features of BMS or atrophic glossitis by nutritional deficiencies, but severe stimulated pain is a pain characteristic of the presence of *Candida* hyphae [24]. Another difference is that if eating increases tongue pain, then it is associated with *Candida*, whereas a decrease in pain while eating is associated with BMS. If there is no difference during eating, then it is a mixed condition [30].

### 4.3. Staphylococcus Aureus Infections

Research shows that not only has *C. albicans* been associated with BMS, but so has *S. aureus* [25]. In our study, *S. aureus* was the second most common cause of infection among patients initially diagnosed with BMS, affecting 13.9% of the patients and 30.8% in the control group.

*S. aureus* is a frequent isolate found in the oral cavity and perioral region. *S. aureus* itself does not play a role in periodontal pathology and can be associated with periodontal health, but since it is known to cause infective endocarditis, this could imply the possibility of the dissemination of the pathogen. Overall, it is not a colonizer of the oral cavity, but a transient microflora [29,31,32].

Non-oral bacteria are not passive bystanders, and could play an essential role in oral and systemic diseases [11].

### 4.4. Klebsiella Species Infections

In our study, *K. pneumoniae* was the most common cause for *Klebsiella* infection among patients with BMS, affecting 8.7% of the patients and 15.1% of the control group.

Research has noted oral infections caused by *Klebsiella* among patients with BMS, which was also found in our study. Additionally, *Klebsiella* species may play a role in the comorbidity of periodontitis and inflammatory bowel disease [25,33].

### 4.5. Enterobacter Infections

In our study, 10.4% of patients diagnosed initially with BMS were affected by *Enterobacter*, but none in the control group.

*Enterobacter* is one of the pathogens that has been found in high frequencies among patients with BMS [25]. In our study, although it was not the most common pathogen, it still had the 5% threshold of infection rates among BMS patients.

*Enterobacter* species are among the Gram-negative pathogens that are a part of the ESKAPE (antimicrobial-resistant) group. They are able to evade the activity of numerous antibacterial agents, including antibiotics, disinfectants, and biocides [22,34].

### 4.6. Non-Fermenting Gram-Negative Rods Infections

In our study, 6.9% of patients diagnosed initially with BMS were affected by non-specific non-fermenting Gram-negative (Gr−) rods, with 4% showing rich growth levels. In the control group, only one individual showed the presence of *A. baumanii*.

Gram-negative bacteria present a high resistance towards antibiotics, and have a great clinical importance, since they put patients in the intensive care unit (ICU) and can lead to high morbidity and mortality [35]. Non-fermenter Gram-negative bacilli (BNF) cause severe, fatal infections, especially in the hospital environment. The main BNF microorganisms that cause disease in humans are *Pseudomonas aeruginosa*; *Acinetobacter baumannii*; *Burkholderia cepacia*; *Stenotrophomonas*; and *Alcaligenes*, *Moraxella.* These stand out for being aerobic and non-sporulated; they are incapable of fermenting sugars [35]. In our study, we found these organisms in patients with BMS (see Table 1 and Table 2).

### 4.7. Escherichia coli Infections

In our study, *E. coli* affected 5.2% of BMS patients, but none of the patients in the control group.

*Escherichia coli* is a common gut bacterium that, when exposed to oxygen, can increase its numbers. Usually, it is outnumbered by anaerobic bacteria. *E. coli* can survive the low pH of the human gastric stomach. Although most strains of *E. coli* do not cause disease, some can produce toxins and other virulent pathogens [36]. Considering the *E. coli* presence in our study in patients with BMS, patients should be observed for oral lichen planus, since some studies have noted that *E. coli* has been observed in oral keratinocytes in oral lichen planus tissue, showing that *E. coli* may play a role in its pathogenesis [37]. 

### 4.8. Other Infections

The rest of the pathogens constituted less than 5% of the affected BMS patients (see Table 1). We can see in our study that the microflora is much richer in patients with BMS compared to healthy people. The small amount of microorganisms diagnosed in some patients with BMS cannot cause specific oral symptoms, and their presence is more likely related with low salivary flow levels.

### 4.9. Limitations of the Study, Implications of the Study for Clinicians, and the Importance of the Study for Researchers

One limitation of the study is the fact that there was a small number of specific pathogens, hindering the possibility of fully seeing the association between the pathogen and BMS, which can be attributed to the smaller sample of patients who were available during the peak of the COVID-19 pandemic.

This study shows that patients who arrive with BMS should always receive a cotton swab test to exclude any possibility of infection, due to the fact that many pathogens can be found among patients. Even though, in this study, we did not find a direct association between infection and BMS symptoms, clinicians should think about unstimulated and stimulated salivary flow levels, since salivary gland disfunction can play a role in an increase in the presence of oral pathogens in the oral cavity. Clinicians should also take into account the patient’s age, since older patients tend to show lower salivary flow rates and higher growth levels of specific oral pathogens.

This study is important to researchers due to the fact that oral pathogens might play a smaller-than-expected role in increases in BMS symptoms. Further research should be performed to determine how the BMS pain/burning levels, salivary flow, and bacterial growth levels change within a certain timeframe, with patients requiring multiple visits.

## 5. Conclusions

In this study, *C. albicans* was the most common pathogen found among patients with BMS. Patients infected with *Enterobacter* species showed the highest and richest growth levels among patients with BMS.

Although patients with present bacterial or *Candida* infections showed marginal increases in pain/burning levels, no direct statistically significant associations could be made between the presence of *Candida* species or other bacteria and the pain/burning levels and salivary flow levels among patients with BMS.

## Figures and Tables

**Table 1:** BMS patients and control group individuals affected by infection.

Pathogen	% of BMS Patients Affected (Number of Patients Affected)	% of Control Group Affected (Number of Persons Affected)
*Candida albicans*	16.2 (28)	23.1 (3)
*Staphylococcus aureus*	13.9 (24)	30.8 (4)
*Enterobacter species*	10.4 (18)	-
*Klebsiella pneumoniae*	8.7 (15)	-
Non-fermenting Gram-negative rods	6.9 (12)	-
*Klebsiella oxytoca*	5.8 (10)	-
*Candida* species	5.2 (9)	7.7 (1)
*Escherichia coli*	5.2 (9)	-
*Klebsiella* species	4.0 (7)	15.1 (3)
*Serratia Marcescens*	2.9 (5)	-
*Acinetobacter lwoffi*	1.7 (3)	-
*Citrobacter* species	1.7 (3)	-
*Acinetobacter baumanii*	1.7 (3)	7.7 (1)
*Aeromonas* species	1.2 (2)	-
*Acinetobacter ursungii*	1.2 (2)	-
*Citrobacter freundii*	1.2 (2)	-
*Raoultella planticola*	1.2 (2)	-
*Pseudomonas fluorescens*	1.2 (2)	-
*Pseudomonas* species	0.6 (1)	-
*Serratia* species	0.6 (1)	-
*Acinetobacter junii*	0.6 (1)	-
*Serratia ficaria*	0.6 (1)	-
*Raoutella ornithinolytica*	0.6 (1)	-
*Pseudomonas putida*	0.6 (1)	-
*Comamonas testosteroni*	0.6 (1)	-
*Burkholderia gladioli*	0.6 (1)	-
*Pantoea* species	0.6 (1)	7.7 (1)
*Sohingomonas paucimobillis*	0.6 (1)	-
*Rahnella aquatilis*	0.6 (1)	-
*Moraxella* species	0.6 (1)	-
*Morganella morganii*	0.6 (1)	-
*Pseudomonas aeroginosa*	0.6 (1)	-
*Enterobacter asburiae*	0.6 (1)	-
*Stenotrophomonas maltophilia*	0.6 (1)	7.7 (1)
*Proteus vulgaris*	0.6 (1)	-

**Table 2:** Growth levels as a percentage (%) among infected BMS patients.

Pathogen	Growth Levels (%) among BMS Patients			Growth Levels (%) among Control Patients		
	Poor	Medium	Rich	Poor	Medium	Rich
*Enterobacter* species	0.6	2.3	7.5	-	-	-
*Klebsiella pneumoniae*	1.7	2.3	4.6	-	-	-
Non-fermenting Gram-negative rods	0	2.9	4	-	-	-
*Escherichia coli*	0.6	1.2	3.5	-	-	-
*Klebsiella oxytoca*	1.7	1.7	2.3	-	-	-
*Candida albicans*	7.5	6.9	1.7	15.4	7.7	0
*Klebsiella* species	1.7	0.6	1.7	7.7	7.7	-
*Staphylococcus aureus*	10.4	2.3	1.2	30.7	0	0
*Candida* species	1.7	2.3	1.2	7.7	0	0
*Serratia Marcescens*	1.7	0	1.2	-	-	-
*Acinetobacter lwoffi*	0.6	0	1.2	-	-	-
*Citrobacter* species	0	0.6	1.2	-	-	-
*Acinetobacter baumanii*	0	0.6	1.2	0	0	7.7
*Citrobacter freundii*	0	0	1.2	-	-	-
*Raoultella planticola*	0	0	1.2	-	-	-
*Pseudomonas fluorescens*	0	0	1.2	-	-	-
*Aeromonas* species	0	0.6	0.6	-	-	-
*Acinetobacter ursungii*	0	0.6	0.6	-	-	-
*Pseudomonas species*	0	0	0.6	-	-	-
*Serratia ficaria*	0	0	0.6	-	-	-
*Raoutella ornithinolytica*	0	0	0.6	-	-	-
*Pseudomonas putida*	0	0	0.6	-	-	-
*Comamonas testosteroni*	0	0	0.6	-	-	-
*Pantoea* species	0	0	0.6	0	7.7	0
*Moraxella* species	0	0	0.6	-	-	-
*Morganella morganii*	0	0	0.6	-	-	-
*Pseudomonas aeroginosa*	0	0	0.6	-	-	-
*Enterobacter asburiae*	0	0	0.6	-	-	-
*Stenotrophomonas maltophilia*	0	0	0.6	0	7.7	0
*Serratia* species	0	0.6	0	-	-	-
*Acinetobacter junii*	0	0.6	0	-	-	-
*Burkholderia gladioli*	0	0.6	0	-	-	-
*Sohingomonas paucimobillis*	0	0.6	0	-	-	-
*Rahnella aquatilis*	0	0.6	0	-	-	-
*Proteus vulgaris*	0	0.6	0	-	-	-

**Table 3:** Differences between infected and non-infected BMS groups (overall infected patients and *C.albicans*-infected patients).

		Infected and Non-Infected BMS Groups			*C. albicans*-Infected and Non-Infected BMS Groups		
		Infected (*n* = 63)	Not Infected (*n* = 110)	Mann–Whitney U Test (*p* = 0.05)	Infected with *C. albicans* (*n* = 28)	Not Infected with C. *albicans* (*n* = 145)	Mann–Whitney U Test (*p* = 0.05)
**Pain levels (median (IQR)) ^1^**	**morning**	2 (IQR 2)	2 (IQR 2)	z = −0.25, *p* = 0.80	2 (IQR 2)	2 (IQR 2)	z = −0.437 *p* = 0.66
	**afternoon**	4 (IQR 2.1)	4 (IQR 2)	z= −0.63, *p* = 0.53	3.5 (IQR 2.3)	4 (IQR 2)	z = 1.130 *p* = 0.26
	**evening**	5 (IQR 2.9)	6 (IQR 3)	z = –1.91 *p* = 0.57	5 (IQR 2)	6 (IQR 3)	z = −0.94 *p* = 0.35
**Salivary flow (median (IQR))**	**Unstimulated**	0.2 (IQR 0.23)	0.2 (IQR 0.3)	z = −0.37 *p* = 0.71	0.18 (IQR 0.15)	0.2 (IQR 0.27)	z = −0.71 *p* = 0.48
	**Stimulated**	0.88 (IQR 0.69)	1 (IQR 0.78)	z = −0.16 *p* = 0.87	0.9 (IQR 0.35)	0.92 (IQR 0.77)	z = −0.262 *p* = 0.79
**Duration of BMS (median (IQR))**	**In months**	5 (IQR 9)	5 (IQR 5)	z = −0.002 *p* = 1.00	6.5 (IQR 9)	5 (IQR 6.5)	z = −0.456 *p* = 0.65

^1^—Used self-explanatory visual analogue scale (VAS) scores from 0–10.

**Table 4:** Differences between infected and non-infected control groups (overall infected patients and *C. albicans*-infected patients).

		Overall Infected and Non-Infected Control Groups			*C. albicans*-Infected and Non-Infected Control Groups		
		Infected (*n* = 8)	Not Infected (*n* = 5)	Mann–Whitney U Test (*p* = 0.05)	Infected with *C. albicans* (*n* = 3)	Not Infected with *C. albicans* (*n* = 10)	Mann–Whitney U Test (*p* = 0.05)
**Salivary flow (median (IQR))**	**Unstimulated**	0.34 (IQR 0.18)	0.32 (IQR 0.7)	z = −0.66 *p* = 0.51	0.28 (IQR 0)	0.34 (IQR 0.26)	z = 1.018 *p* = 0.31
	**Stimulated**	1.35 (IQR 1.34)	1 (IQR 0.9)	z = −1.03 *p* = 0.30	1.2 (IQR 0)	1.2 (IQR 1.05)	z = −0.00 *p* = 1

## Data Availability

The data are not publicly available due to privacy concerns.

## Supplementary Materials

The following supporting information can be downloaded at: https://www.mdpi.com/1648-9144/58/8/1029/s1. Table S1. Differences between infected and non infected BMS groups (showing all pathogens exceeding 5% of infection rates); Table S2 Correlations between BMS groups patient data; Table S3 Correlations between Control groups patient data; Table S4: Differences between infected and non infected control groups regarding all pathogens.
