# Supplementary material for: Candidiasis and Other Bacterial Infections among Patients Diagnosed with Burning Mouth Syndrome
Source: Medicina (Kaunas). 2022 Aug 1;58(8):1029. doi: 10.3390/medicina58081029 (PMC9416425; doi:10.3390/medicina58081029)
Supplement: Supplementary file 1 [file medicina-58-01029-s001.zip › Table S1.pdf]

Table S1. Differences between infected and non infected BMS groups (showing all pathogens exceeding 5% of infection rates)

		**Infected and non-infected BMS groups**			***C.albicans* infected and non-infected BMS groups**			***S. aureus* infected and non-infected BMS groups**		
		**Infected (n= 63)**	**Not infected (n= 140)**	**Mann Whitney U test (p=0.05)**	**Infected with C. albicans (n=28)**	**Not infected with C. albicans (n=145)**	**Mann Whitney U test (p=0.05)**	**Infected with S. aureus (n=24)**	**Not infected with S. aureus (n=149)**	**Mann Whitney U test (p=0.05)**
**Pain levels (median (IQR))**	**Morning**	2 (IQR 2)	2 (IQR 2)	z=-0.25, p=0.80	2 (IQR 2)	2 (IQR 2)	z=-0.44<br>p=0.66	2 (IQR 2)	2 (IQR 2)	z=-0.80<br>p=0.42
	**Afternoon**	4 (IQR 2.1)	4 (IQR 2.0)	z = -0.63,<br>p = 0.53	3.5 (IQR 2.3)	4 (IQR 2)	z=1.13<br>p=0.26	4 (IQR 1)	4 (IQR 2)	z=-1.20<br>p=0.23
	**Evening**	5 (IQR 2.9)	6 (IQR 3)	z = –1.91<br>p= 0.57	5 (IQR 2)	6 (IQR 3)	z=-0.94<br>p=0.35	5.75 (IQR 2.4)	5.5 (IQR 3.0)	z=-0.27<br>p=0.79
**Salivary flow levels (median (IQR))**	**Unstimulated**	0.2 (IQR 0.23)	0.2 (IQR 0.3)	z = -0.37<br>p= 0.71	0.18 (IQR 0.15)	0.2 (IQR 0.27)	z=-0.71<br>p=0.48	0.16 (IQR 0.27)	0.2 (IQR 0.24)	z=-1.31<br>p=0.19
	**Stimulated**	0.88 (IQR 0.69)	1 (IQR 0.78)	z = -0.16<br>p= 0.87	0.9<br>(IQR 0.35)	0.92 (IQR 0.77)	z=-0.26<br>p=0.79	1 (IQR 0.56)	0.9 (IQR 0.76)	z=-0.26<br>p=0.79
**Duration of BMS (median (IQR))**	**In months**	5 (IQR 9)	5 (IQR 5)	z = -0.002<br>p= 1.00	6.5 (IQR 9)	5 (IQR 6.5)	z=-0.46<br>p=0.65	4 (IQR 9)	6 (IQR 9)	z=-0.68<br>p=0.50

		***Enterobacter species* infected and non-infected BMS groups**			***Klebsiella pneumoniae* infected and non-infected BMS groups**			**Non fermenting Gram negative rods infected and non-infected BMS groups**		
		**Infected with *Enterobacter species* (n=18)**	**Not infected with *Enterobacter species* (n=155)**	**Mann Whitney U test (p=0.05)**	**Infected with *Klebsiella pneumoniae* (n=15)**	**Not infected with *Klebsiella pneumoniae* (n=158)**	**Mann Whitney U test (p=0.05)**	**Infected with Non fermenting Gram negative rods (n=12)**	**Not infected with Non fermenting Gram negative rods (n=161)**	**Mann Whitney U test (p=0.05)**
**Pain levels (median (IQR))**	**Morning**	2 (IQR 3.3)	2 (IQR 2)	z=-0.18<br>p=0.86	2 (IQR 1)	2 (IQR 2)	z=-0.42<br>p=0.97	2 (IQR 1.4)	2 (IQR 2)	z=-1.1<br>p=0.27
	**Afternoon**	4.25 (IQR 2.3)	4 (IQR 2)	z=-1.12<br>p=0.26	4 (IQR 2)	4 (IQR 1)	z=-0.18<br>p=0.86	3.75 (IQR 1)	4 (IQR 2)	z=-0.59<br>p=0.55
	**Evening**	6 (IQR 6)	5.5 (IQR 3)	z=-0.20<br>p=0.85	5.75 (IQR 3)	5 (IQR 4)	z=-0.69<br>p=0.49	5.75 (IQR 4.8)	5.5 (IQR 3)	z=-0.44<br>p=0.66
**Salivary flow levels (median (IQR))**	**Unstimulated**	0.18 (IQR 0.32)	0.2 (IQR 0.2)	z=-0.61<br>p=0.54	0.2 (IQR 0.29)	0.2 (IQR 0.28)	z=-0.52<br>p=0.61	0.24 (IQR 0.32)	0.2 (IQR 0.23)	z=-0.58<br>p=0.57
	**Stimulated**	0.6 (IQR 0.54)	0.96 (IQR 0.68)	z=-1.83<br>p=0.68	0.8 (IQR 0.56)	0.96 (IQR 0.74)	z=-1.4<br>p=0.16	0.93 (IQR 0.61)	0.9 (IQR 0.74)	z=-0.28<br>p=0.78
**Duration of BMS (median (IQR))**	**In months**	5.5 (IQR 6.0)	5 (IQR 9)	z=-0.01<br>p=0.99	6 (IQR 8.5)	9 (IQR 5)	z=-0.33<br>p=0.97	5 (IQR 9)	5 (IQR 9)	z=-0.29<br>p=0.77

		***Klebsiella oxytoca* infected and non-infected BMS groups**			***Candida species* infected and non-infected BMS groups**			***Escherichia coli* infected and non-infected BMS groups**		
		**Infected with *Klebsiella oxytoca* (n=10)**	**Not infected with *Klebsiella oxytoca* (n=163)**	**Mann Whitney U test (p=0.05)**	**Infected with *Candida species* (n=9)**	**Not Infected with *Candida species* (n=164)**	**Mann Whitney U test (p=0.05)**	**Infected with *Escherichia coli* (n=9)**	**Not infected with *Escherichia coli* (n=164)**	**Mann Whitney U test (p=0.05)**
**Pain levels (median (IQR))**	**Morning**	4 (IQR 2.5)	2 (IQR 2)	z=-1.81<br>p=0.07	2 (IQR 2)	2 (IQR 2)	z=-0.37<br>p=0.71	2 (IQR 3.5)	2 (IQR 2)	z=-0.'05<br>p=0.96
	**Afternoon**	5 (IQR 4)	4 (IQR 2)	z=-1.16<br>p=0.25	2 (IQR 2.5)	4 (IQR 2)	z=-2.12<br>p=0.03	5 (IQR 4)	4 (IQR 2)	z=-0.54<br>p=0.59
	**Evening**	4 (IQR 5.3)	5.5 (IQR 3)	z=-1.1<br>p=0.29	4 (IQR 5)	5.5 (IQR 3)	z=-0.74<br>p=0.46	5 (IQR 2.5)	6 (IQR 3)	z=-0.16<br>p=0.88
**Salivary flow levels (median (IQR))**	**Unstimulated**	0.24 (IQR 0.45)	0.2 (IQR 0.22)	z=-0.14<br>p=0.89	0.2 (IQR 0.12)	0.2 (IQR 0.27)	z=-0.64<br>p=0.52	0.2 (IQR 0.6)	0.2 (IQR 0.23)	z=-1.03<br>p=0.31
	**Stimulated**	1.02 (IQR 1.09)	0.9 (IQR 0.72)	z=-0.74<br>p=0.46	0.76 (IQR 0.98)	0.94 (IQR 0.72)	z=-1.39<br>p=0.17	1.28 (IQR 1.59)	0.9 (IQR 0.7)	z=-1.13<br>p=0.26
**Duration of BMS (median (IQR))**	**In months**	6 (IQR 9)	5 (IQR 9)	z=-0.16<br>p=0.99	4 (IQR 21)	5.5 (IQR 9)	z=-0.86<br>p=0.93	5 (IQR 7.75)	5 (IQR 9)	z=-0.96<br>p=0.92
