# Supplementary material for: Candidiasis and Other Bacterial Infections among Patients Diagnosed with Burning Mouth Syndrome
Source: Medicina (Kaunas). 2022 Aug 1;58(8):1029. doi: 10.3390/medicina58081029 (PMC9416425; doi:10.3390/medicina58081029)
Supplement: Supplementary file 1 [file medicina-58-01029-s001.zip › Table S4.pdf]

Table S4. Differences between infected and non infected control groups regarding all pathogens.

		Overall infected and non-infected control groups			*C.albicans* infected and non-infected control groups			*S. aureus* infected and non-infected control groups			*Klebsiella species* infected and non-infected control groups		
		**Infected (n= 8)**	**Not infected (n= 5)**	**Mann Whitney U test (p=0.05)**	**Infected with C. albicans (n=3)**	**Not infected with C. albicans (n=10)**	**Mann Whitney U test (p=0.05)**	**Infected with S. aureus (n=4)**	**Not infected with S. aureus (n=9)**	**Mann Whitney U test (p=0.05)**	**Infected with *Klebsiella species* (n=2)**	**Not infected with *Klebsiella species* (n=11)**	**Mann Whitney U test (p=0.05)**
**Salivary flow (median (IQR))**	**Unstimulated**	0.34 (IQR 0.18)	0.32(IQR 0.7)	z= -0.66<br>p= 0.51	0.28(IQR 0)	0.34(IQR 0.26)	z=-1.02<br>p=0.31	0.35(IQR 0.54)	0.32(IQR 0.18)	z=-0.31<br>p=0.76	0.33	0.32(IQR 0.2)	z=-0.40<br>p=0.77
	**Stimulated salivary**	1.35 (IQR 1.34)	1 (IQR 0.9)	z= -1.03<br>p= 0.3	1.2<br>(IQR 0)	1.2 (IQR 1.05)	z=-0.0<br>p=1	1.65(IQR 2.39)	1.2(IQR 0.9)	z=-1.09<br>p=0.28	1.31	1.2(IQR 0.9)	z=0<br>p=1
